# Ancient RNA stems that terminate transcription

**DOI:** 10.4161/rna.28342

**Published:** 2014-03-05

**Authors:** Nikolay Zenkin

**Affiliations:** Centre for Bacterial Cell Biology; Institute for Cell and Molecular Biosciences; Newcastle University; Baddiley-Clark Building; Newcastle upon Tyne, UK

**Keywords:** RNA polymerase, transcription, termination, RNA hairpin, RNA secondary structure, elongation complex, pausing, molecular evolution, Last Universal Common Ancestor

## Abstract

Multi-subunit RNA polymerases are the enzymes that perform transcription in all living organisms and that have emerged before the divergence of domains of life. The structures of catalytic cores and their functions during elongation step of transcription cycle are very similar for all multi-subunit RNA polymerases. In contrast, the mechanisms for terminating the RNA synthesis have seemingly diverged in modern RNA polymerases. However, the recent finding that, much like during bacterial transcription, RNA secondary structure is involved in termination by eukaryotic RNA polymerase III (pol III), suggests that RNA-dependent termination may have emerged before the divergence of bacterial and archaeal/eukaryotic RNA polymerases. In the case of pol III, the terminating RNA secondary structures are not dedicated hairpins, but are formed by the bodies of highly structured transcripts, which are clearly the remnants from the RNA–protein world. Here I discuss the similarities and differences of RNA-dependent mechanisms of termination of transcription by bacterial RNA polymerase and pol III.

Termination is an obligatory event that causes extremely stable transcription elongation complex to disassemble at the end of the gene with the release of RNA polymerase (RNAP) and the transcript from the template DNA. Termination is required for proper expression of neighboring genes, maturation and export of transcripts (in eukaryotes), recycling of RNAP, and clearing the template for subsequent transcription. Though mechanisms involved in transcription elongation are very similar for bacterial RNAP, archaeal RNAP and pol I, pol II, and pol III from eukaryotes (plant-specific RNAPs IV and V are not discussed here), the mechanisms of transcription termination seem to be strikingly different (see below). However, recently, we showed that, similarly to termination in bacteria, termination by eukaryotic pol III involves formation of RNA secondary structure,^1^ suggesting that the RNA-dependent termination may have been the primordial mechanism used by the common ancestor of multi-subunit RNAPs.

In bacteria, destruction of the elongation complex during termination is facilitated by a dedicated > 7 base pairs-long G:C-rich RNA hairpin that folds behind RNAP.^2^^,^^3^ Termination by pol III is also caused by RNA secondary structure.^1^ However, in this case, the RNA secondary structure comes from the body of the synthesized RNA. Pol III transcribes genes of structural and catalytic RNAs (5S, SRP, RNase MRP, RNase P, U6 RNAs, tRNAs), which, as per their functions, have extensive secondary/ternary structures. The terminating RNA stem on these genes can be formed by distant parts of the transcript, such as the acceptor stem of the tRNA, formed by the very 5′ and 3′ proximal parts of the molecule (Fig. 1A).

**Figure:**
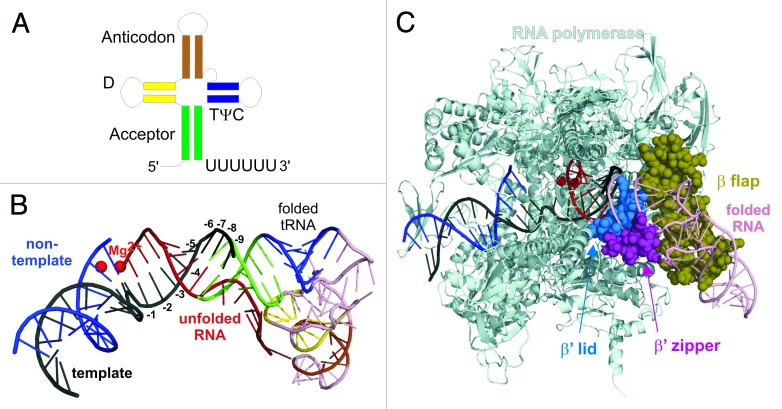
**Figure 1.** RNA secondary structure-dependent termination of transcription. (**A**) Scheme of the tRNA secondary structure. Note that both acceptor stem and TψC stem-loop can serve as termination secondary structures depending on the extent of pol III backtracking on the oligoT signal (see text). (**B**) Nucleic acids scaffold in the elongation complex (pdbid: 2PPB).^14^ Mg^2+^ ions of the active center are shown as red spheres. Template and non-template DNA are black and dark blue, respectively; RNA is red. A pol III transcript, represented here by tRNA molecule (pdbid: 1EHZ),^15^ folded at the distance sufficient for termination, is color coded as in panel **A** (with loops in pink). Note the interference of 5′ end nucleotides of the tRNA with the template DNA bases at positions 8th and 9th in the RNA–DNA hybrid. The real orientation of the folded tRNA relative to the hybrid may be different. More base pairs of the hybrid could be melted by the folded tRNA due to the collision of tRNA with protein domains, which could exert a pulling force on the hybrid. (**C**) Interference of the folded tRNA (pink) termination structure with domains of RNAP (cyan ribbon). β flap, β’ zipper, and β’ lid are shown as khaki, magenta and blue spheres, respectively. Relative orientation of tRNA as in panel **B**. The real orientation may differ, leading to collisions with even more domains of RNAP.

Despite this apparent difference, the mechanisms of termination between bacterial RNAP and pol III appear to be very similar. As seen from Figure 1B, the 5′ end (5′ end shoulder of the acceptor stem) of the folded tRNA interferes with the template DNA strand in the RNA–DNA hybrid. This may shorten the hybrid, which is the major determinant of the stability of the elongation complex, to a critical length of 7 bp (from the RNAP active center), when the elongation complex loses its stability.^4^ If the RNA stem is just 1 bp shorter, permitting 8 bp hybrid, the termination becomes much less efficient,^1^ consistent with observations that elongation complexes with such hybrid length are stable.^4^ A very similar situation was observed for termination by bacterial RNAP.^5^^,^^6^ Note that shortening of the RNA–DNA hybrid may also happen as a result of a sterical clash of the RNA stem and the rear end of the elongation complex, which may lead to a force pulling on the RNA in the hybrid (rather than interference of the stem with the RNA–DNA hybrid), as was shown for bacterial termination.^5^ However, the collision of the forming acceptor stem (in the case of tRNA) with structural elements of the elongation complex may play a more critical role in destabilization of the complex, as was proposed for bacterial termination.^6^^,^^7^ A termination stem/hairpin would sterically clash with at least β flap, β’ zipper, and β’ lid (Fig. 1C).^7^ Furthermore, the interference with the 5′ end of the RNA–DNA hybrid (via displacement or pulling) would lead to disruption of multiple protein–hybrid contacts, such as the ones with β’ rudder, which stabilize the elongation complex.^7^^,^^8^

The ubiquitous hairpins that form during elongation cannot destruct the actively transcribing elongation complex.^2^ For termination to occur, RNAP has to pause, allowing the termination hairpin to form.^6^ Besides the similarities and clear involvement of the RNA secondary structure in termination, there are some noticeable differences in pre-termination pausing between bacterial and pol III enzymes. The most apparent difference is the role of oligoT (in non-template DNA strand) tract downstream of RNA secondary structures. In bacteria, the oligoT tract serves to briefly pause RNAP by causing short backtracking, which allows sufficient time for the hairpin formation.^6^^,^^7^ In addition, the U:A RNA–DNA hybrid in this paused complex is required for efficient complex destruction, apparently due to its relative instability.^2^^,^^5^ During pol III termination, oligoT tract also pauses transcription and forces pol III into backtracking. However, in this case, backtracking appears to be almost irreversible and, if continues beyond four to five nucleotides, leads to catalytic inactivation of pol III.^1^ In a backtracked complex, addition of NMPs is blocked since the 3′ end of RNA has disengaged from the catalytic site. However, quite surprisingly, the highly efficient RNA hydrolysis activity, which in pol III is stimulated by the C11 subunit and would rescue the backtracked pol III by restoring the 3′ end in the active center, is also inhibited.^1^ Switching off of the cleavage activity could be explained by sterical replacement of C11 from the active center by the extruding 3′ end of backtracked RNA. Such a complete catalytic inactivation, in contrast to transient pausing in bacteria, commits the elongation complex to termination.

Another unusual property of termination of pol III transcription is the insensitivity to the RNA–DNA hybrid sequence. Backtracking on the oligoT signal can shift pol III backward as far as ~12 bp, replacing the U:A-rich hybrid in the elongation complex with a sequence that precedes it in the gene.^1^ This, however, does not affect destruction of the complex by the hairpin, suggesting a generally lower stability of the pol III elongation complexes as compared with complexes of bacterial RNAP or a lesser importance of hybrid shortening during pol III termination. Extensive backtracking highlights yet another unique feature of termination by pol III. In contrast to bacteria, where the terminating RNA hairpin has to form at a strictly defined distance from the active site of RNAP,^9^ the RNA stem of pol III transcripts can be as far as 12 nucleotides upstream of the oligoU stretch.^1^ Accordingly, in genes transcribed by pol III, this distance varies from 0–12 nucleotides (in *S. cerevisiae*).^1^ On some genes with short distances, pol III may use for termination not the nearest but the second nearest RNA secondary structure of its highly structured transcripts, such as TψC stem-loop that precedes the acceptor stem in tRNA (Fig. 1A; Fig. S8 in ref. 1). Such flexibility possibly increases the chances for efficient termination by pol III if the ultimate RNA hairpin fails to fold for some reason. In the case of bacterial RNAP, extensive backtracking on a termination signal would not be beneficial given that the majority of transcribed genes are coding for mRNAs, which are unlikely to have secondary structures just behind the dedicated termination hairpins.

Based on the structural similarities between the mechanisms of termination by bacterial RNAP and eukaryotic pol III, the requirement for the RNA stem for destruction of the elongation complex and the distance between this stem and the RNAP active center, it can be hypothesized that the RNA stem-dependent termination of transcription emerged before divergence of bacteria and archaea/eukaryotes. Pol II was shown to be able to terminate transcription in RNA hairpin-dependent manner in vitro,^5^ and archaeal RNAP may require RNA stem folding for termination.^1^^,^^10^ Pol I is also known to efficiently terminate synthesis of its highly structured transcript in a factor-independent manner in vitro.^11^ Together, these observations are consistent with the possibility that RNA hairpin-dependent termination may have been the primordial mechanism used to stop transcription in the Last Universal Common Ancestor (LUCA), although later it may have been replaced by the protein factors. The early emergence of RNAP puts it at the stage of evolution that was dominated by ribozymes and structural RNAs (such as ribosomal RNAs or tRNAs that survived till the present days) characterized by extensive secondary structures. It seems reasonable to suggest that transcription termination caused by the structure of the transcript would be beneficiary for the LUCA; destruction of the elongation complex caused by a fully folded functional RNA would provide a simple factor-independent mechanism for termination of transcription right at the end of the gene. Furthermore, RNA secondary structures affect only paused/stalled elongation complexes (irrespective of the sequence of the duplex), possibly also providing a mechanism for displacement of prematurely stalled complexes.

Though the RNA stem-dependent termination may have been the ancestral mechanism, it apparently has been supplemented and/or displaced by protein-dependent mechanisms after the divergence of multi-subunit RNAPs. Bacteria, in addition to the hairpin-dependent intrinsic termination, acquired protein-mediated termination that uses an RNA helicase ρ or a DNA translocase Mfd to destruct the elongation complex. Pol I and Pol II have acquired mechanisms involving RNA exonucleases and/or an RNA helicase,^12^^,^^13^ which seemingly replaced RNA-dependent termination. It is likely that the archaeal RNAP may also require accessory termination proteins, and pol III may have a protein-assisted back-up mechanism in addition to the RNA-dependent termination. The two possible reasons for the emergence of new termination strategies are the growing demands for the efficiency of transcription and, as a result, for its termination; and a shift from highly structured RNAs to protein-coding mRNAs that are largely devoid of regular secondary/ternary structures that could facilitate termination.

## Notes

10.4161/rna.28342
